# Investigating the metabolic capabilities of *Mycobacterium tuberculosis *H37Rv using the *in silico *strain *iNJ*661 and proposing alternative drug targets

**DOI:** 10.1186/1752-0509-1-26

**Published:** 2007-06-08

**Authors:** Neema Jamshidi, Bernhard Ø Palsson

**Affiliations:** 1 Department of Bioengineering, University of California, San Diego, La Jolla, CA, 92093-0412, USA.

## Abstract

**Background:**

*Mycobacterium tuberculosis *continues to be a major pathogen in the third world, killing almost 2 million people a year by the most recent estimates. Even in industrialized countries, the emergence of multi-drug resistant (MDR) strains of tuberculosis hails the need to develop additional medications for treatment. Many of the drugs used for treatment of tuberculosis target metabolic enzymes. Genome-scale models can be used for analysis, discovery, and as hypothesis generating tools, which will hopefully assist the rational drug development process. These models need to be able to assimilate data from large datasets and analyze them.

**Results:**

We completed a bottom up reconstruction of the metabolic network of *Mycobacterium tuberculosis *H37Rv. This functional *in silico *bacterium, *iNJ*661, contains 661 genes and 939 reactions and can produce many of the complex compounds characteristic to tuberculosis, such as mycolic acids and mycocerosates. We grew this bacterium *in silico *on various media, analyzed the model in the context of multiple high-throughput data sets, and finally we analyzed the network in an 'unbiased' manner by calculating the Hard Coupled Reaction (HCR) sets, groups of reactions that are forced to operate in unison due to mass conservation and connectivity constraints.

**Conclusion:**

Although we observed growth rates comparable to experimental observations (doubling times ranging from about 12 to 24 hours) in different media, comparisons of gene essentiality with experimental data were less encouraging (generally about 55%). The reasons for the often conflicting results were multi-fold, including gene expression variability under different conditions and lack of complete biological knowledge. Some of the inconsistencies between *in vitro *and *in silico *or *in vivo *and *in silico *results highlight specific loci that are worth further experimental investigations. Finally, by considering the HCR sets in the context of known drug targets for tuberculosis treatment we proposed new alternative, but equivalent drug targets.

## Background

Tuberculosis continues to be a devastating pathogen throughout the world, particularly in developing nations. In 2001, the World Health Organization (WHO) estimated 8.5 million new cases of tuberculosis (based on 3.8 million new reported cases) and an estimated 1.8 million deaths from tuberculosis in 2000 [1]. Within the United States, the number of reported cases of tuberculosis has been decreasing with the exception of a period when the trend reversed in 1986 and peaked in 1992 [2,3]. This reversal has been attributed principally to HIV/AIDs, immigration from countries with high prevalence of tuberculosis, poverty, homelessness, and multi-drug resistant (MDR) tuberculosis [1,2]. MDR tuberculosis is generally defined as strains that are resistant to treatment with isoniazid and rifampin [4], two of the key first line antituberculosis drugs [1]. MDR strains of tuberculosis emerged in the early 1990s and have now been found all over the world [4].

Many of the unique properties of tuberculosis are attributable to its metabolism, particularly the complex fatty acids characteristics of the organism. These mycolic acids, phenolic glycolipids, and mycoceric acids confer many of the properties such as its acid-fastness and are believed to contribute to the resilience of the organism. *Mycobacterium tuberculosis *can survive in a wide range of environments (many different tissues) and fairly extreme pHs [5]. One of the most confounding factors with these bacteria is their ability to survive for long periods of time in a dormant stage. The slow doubling time of tuberculosis has further limited the amount of experimental data that can be generated. Many of the first and second line drugs used to treat tuberculosis have metabolic targets, so developing systems level models of metabolism are anticipated to be of great use in the future.

DNA sequencing of the ~4.4 Mbp genome of *Mycobacterium tuberculosis *H37Rv (*M. tb*) in 1998 [6] enabled the ability the pursuit of genome-scale analyses of this microorganism. The remarkable relevance to world health and disease control and the need to understand the metabolic function of the organism all evoke the need of a genome-scale metabolic model. Long-term anticipated goals and applications of such models are to understand the growth of mycobacteria under different conditions, identifying strategies to improve growth *in vitro *(for experimental and diagnostic purposes), and identifying new drug targets for treatment.

In order to gain understanding about the unique characteristics of this important pathogen, we manually reconstructed the metabolic network of *M. tb in silico *(*iNJ*661), from which we developed a model to compute perform computational analyses and interpret experimental data. These bottom-up reconstructions have been described in the past as, biochemically, genetically and genomically structured (BiGG) 'databases'. We employ constraint-based reconstruction and analysis (COBRA) of this BiGG reconstruction to learn about its normal metabolic function and to infer new potential targets for drugs.

## Results and discussion

**Figure 1:**
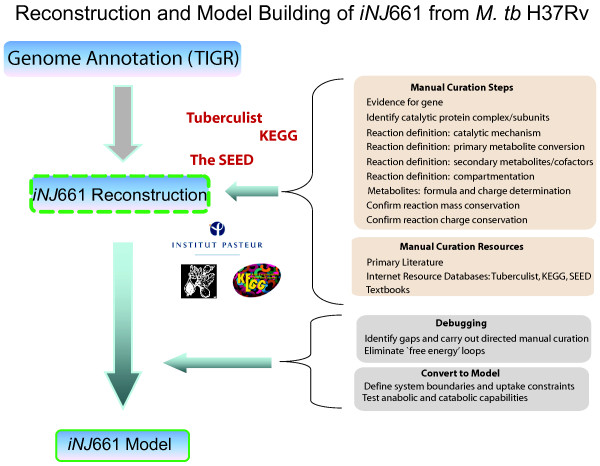
The Gene Index for Mycobacterium tuberculosis H37Rv was downloaded from The Institute for Genomic Research (TIGR) [45]. Reconstruction content was defined based on the sequence annotation, legacy data, the Tuberculist database [31], ancillary sources such as the Kyoto Encyclopedia of Genes and Genomes (KEGG), and SEED [47]. Reactions were defined and according to the Tuberculist web and KEGG. Legacy data was also used in the process of building the model from the reconstruction. The manual curation aspects of the reconstruction are outlined above and discussed in further detail in [42]. Debugging was started once the first draft of the reconstruction was completed and functional testing (i.e. flux balance analysis calculations, etc.) were begun.

The reconstruction process has been described previously [7,8], Figure 1 summarizes this process in brief. The network statistics for *iNJ*661, which has 661 genes and 939 intra-system reactions, are summarized in Table 1. A biomass objective function was defined using available measurements of *M. tb *H37Rv and other mycobacteria strains if information was lacking. The biomass objective function was defined using the literature for chemical composition studies of *M. tb *[9-13]. When such information was not found for the specific strain, *Mycobacterium bovis *was used (for example to approximate the biomass composition of nucleotides and peptidoglycans [14]). The simple fatty acids compositions were based on studies of the mycobacterial cell wall [15]. The biomass function includes 90 metabolites in addition growth associated maintenance ATP costs (see Table 2). [See Additional file 1] for more detailed information.

**Table 1:** The confidence level for each reaction is based on a scale from 0 to 4, 4 being the highest level of confidence (experimental biochemical evidence supporting the inclusion of a reaction) and 1 being the lowest level of confidence (inclusion of a reaction solely on modeling functionality). Sequence based annotations have a confidence level of 2.

**Network properties of *iNJ*661**	
Genes	661
Proteins	543
Reactions (intra-system)	939
Reactions (exchange)	88
Gene Associated Reactions	77%
Metabolites	828
Average Confidence level	2.31

### *M. tb *growth *in silico*

Flux Balance Analysis (FBA) was used to grow *iNJ*661 *in silico *maximizing the biomass function defined in Table 2 (see Materials and Methods). *iNJ*661 was grown *in silico *on three different types of media, Middlebrook 7H9 (supplemented with glucose and glycerol) [16], Youmans [17], and the chemically defined rich culture media (CAMR) [18]. Uptake fluxes which define the different media conditions are shown in Table 3 and the computed results under the different conditions are summarized in Table 4. The doubling times are within the range described in the literature [18,19], and as expected, the growth rates are higher with the richer media. The minimum doubling time of 14.7/hr described by [18] are within the capabilities of maximum growth of *iNJ*661.

**Table 2:** The list of biomass components for the initial biomass function. [See Additional file 1] for the additional components added for the extended biomass function and [see Additional file 5] for the explicit names and molecular formulas.

**Biomass composition for growth of *iNJ*661**		
**mmol/gDW**	**Abbreviation**	

0.40596	ala-L	
0.02261	cys-L	
0.12031	asp-L	
0.09001	glu-L	
0.04810	phe-L	
0.33581	gly	
0.04062	his-L	
0.08773	ile-L	
0.03909	lys-L	
0.20471	leu-L	**amino acids**
0.03489	met-L	
0.04770	asn-L	
0.13803	pro-L	
0.05812	gln-L	
0.12042	arg-L	
0.14340	ser-L	
0.13571	thr-L	
0.20583	val-L	
0.02012	trp-L	
0.03176	tyr-L	

0.13903	amp	
0.25095	cmp	
0.24365	gmp	
0.13160	ump	**nucleic acids**
0.00349	damp	
0.00666	dcmp	
0.00666	dgmp	
0.00365	dtmp	

0.00797	tre	
0.00118	tat	
0.00125	pat	
0.00114	sl1	
0.00649	tre6p	
0.00647	tres	
0.16315	glc-D	
0.09507	man	**sugars**
0.02172	rib-D	**carbohydrates**
0.05547	gal	**peptidoglycans**
0.23018	acgam1p	
0.00279	uamr	
0.00101	uaaAgla	
0.00101	uaaGgla	
0.00099	uaagmda	
0.00098	ugagmda	
0.02536	glyc	
0.01885	peptido_TB1	
0.01885	peptido_TB2	
0.00073	arabinanagalfragund	
0.00168	Ac1PIM1	
0.00149	Ac1PIM2	
0.00134	Ac1PIM3	
0.00121	Ac1PIM4	
0.00127	Ac2PIM2	
0.00208	PIM1	
0.00179	PIM2	
0.00157	PIM3	
0.00140	PIM4	
0.00127	PIM5	
0.00115	PIM6	
0.00406	pe160	
0.00586	clpn160190	**fatty acids**
0.01219	ttdca	**glycolipids**
0.23515	hdca	**phospholipids**
0.01094	hdcea	
0.03484	ocdca	
0.00985	ocdcea	
0.09580	mocdca	
0.01568	arach	
0.00784	mbhn	
0.05835	hexc	
0.00724	pa160	
0.00680	pa160190	
0.00641	pa190190	
0.00649	pg160	
0.00613	pg160190	
0.00581	pg190	

0.01100	mycolate	
0.00334	kmycolate	
0.00329	mkmycolate	
0.00337	mmmycolate	
0.00111	tmha1	**mycolic acids**
0.00110	tmha2	**phenolic glycolipids**
0.00110	tmha3	**mycocerosates**
0.00110	tmha4	**mycobactin**
0.00645	phdca	
0.00118	mfrrppdima	
0.00140	mcbts	
0.00150	fcmcbtt	
0.00027	pdima	
0.00026	ppdima	

60.00000	atp	
60.00000	adp (product)	**growth associated maintenance**
60.00000	pi (product)	

**Table 3:** *Glucose and glycerol supplementation were added as described in [20].

***in silico *media composition**		
**Youmans**	**Middlebrook 7H9**	**CAMR**

oxygen	ammonium	L-alanine
L-asparagine	calcium	L-arginine
citrate	chloride	L-asparagine
glycerol	citrate	L-aspartic acid
water	copper	L-glutamic acid
octadecanoate (Tween)	ferric iron	L-glycine
phosphate	L-glutamate	L-isoleucine
sulfate	magnesium	L-leucine
magnesium	oxygen	L-serine
potassium	phosphate	L-phenylalanine
bicarbonate	sodium	pyruvate
(ferric iron: minimal amount)	sulfate	glycerol
	water	octadecanoate (Tween)
	D-glucose*	
	glycerol*	

**Table 4:** Summary of *iNJ*661 biomass production rates (in mmol/hr/g dwt) and doubling times (1/hr) *in silico *on different media. * from [19], ^ from [18].

**iNJ661 Growth**			
	**Biomass Accumulation**	***iNJ*661 Doubling Time**	***M. tb *Doubling Time**

**Middlebrook 7H9**	0.052	13.33	
**Youman's**	0.029	23.90	24*
**CAMR**	0.058	11.95	14.7^

**Figure 2:**
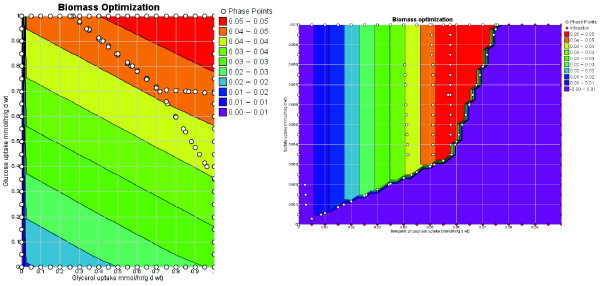
a. Phase plane diagram of glycerol versus glucose uptake while optimizing for growth on Middlebrook media. iNJ661 can grow with glycerol serving as the sole carbon source, but not glucose. The open dots are the calculated phase points and the solid black lines indicate isoclines. b. Phase plane diagram for phosphate versus sulfate uptake on Middlebrook media. Although the transport fluxes are very small, they are both necessary for the organism to be able to grow.

As noted by Primm et al [20], *M. tb *growth on Middlebrook 7H9 media requires supplementation with glucose and glycerol. Glycerol is a component of the biomass function (Table 2) according to the chemical composition measurements performed by [14] on *Mycobacterium bovis*. So in order to grow *in silico*, glycerol will either need to be in the medium or the cell must have some way of producing it from other precursors. *iNJ*661 was able to grow when glucose uptake was abrogated, however, it always required glycerol supplementation in the media which could be due to the biomass requirement or due to the need for glycerol for the production of a different essential metabolite. The growth dependence on glycerol and glucose uptake can be seen in the phase plane diagram depicted in Figure 2a. In this figure, the biomass objective function is optimized while the glucose and glycerol uptake fluxes are varied in different combinations. The resulting plot shows how the growth capability of *iNJ*661 varies as a function of glucose and glycerol uptake. The solid black lines depict isoclines, along which growth is constant [21]. Further investigations were carried out in order to understand the essentiality of glycerol and non-essentiality of glucose in *iNJ*661. Since flux distributions calculated by FBA are non-unique, flux variability analysis (see Methods) can be used to calculate the span of each flux while still achieving the maximum value for the objective function. Reactions with non-zero fluxes, in which the maximum flux equals the minimum flux (and is non-zero), are essential in achieving the particular objective (no alternative pathways exist). Flux variability [22] at maximum growth, with Middlebrook media with glucose and glycerol supplementation (Table 2) was performed in order to identify the constraining flux(es) that prevents growth on glucose but not glycerol. The only non-zero fluxes with no flexibility, directly involving the metabolism of glycerol, were the glycerol transporter (GLYCt) and glycerol kinase (Rv3696c, GlpK, GLYK). The production of glycerol-3-phosphate via glycerol kinase is essential for membrane and fatty acid metabolism involving fatty-acyl glycerol phosphates and interconversions between CDP-diacyl-glycerol and phosphatidyl-glycerol phosphates. These results are consistent and expected given the importance of membrane metabolism in the role of biomass production.

The non-zero fluxes with no flexibility directly involving glucose were the glucose transporter (GLCabc, SugABC, Rv1236+Rv1237+Rv1238) and glucokinase (PPGKr, PpgK, Rv2702). Glucose-6-phosphate is subsequently essential for a number of other reactions involving various aspects of membrane metabolism, including the synthesis of trehalose phosophate (TRE6PS, OtsA, Rv3490) and inositol-phosphate (MI1PS, Ino1, Rv0046c), in addition to phosphoglucomutase (PGMT, PgmA, Rv3068c). These three reactions are required not only for optimal growth, but they are in fact essential for any growth at all *in silico*. Only one of these, however, has been suggested to be essential experimentally (Rv3490) [23]. Growing *iNJ*661 in Middlebrook media (with glycerol but without glucose supplementation) severely retards the growth rate, however it is still viable *in silico *in contrast to *M. tb *growth *in vitro*. The discrepancy between *in vitro *essentiality of glucose, but non-essentiality of reactions requiring glucose derivatives and *in silico *non-essentiality of glucose suggest that there may be non-metabolic functions related to the need for glucose, in addition to alternative metabolic pathways for the production and/or use of glucose-6-phosphate.

Figure 2b characterizes the growth dependence on the uptake rates of inorganic phosphate and sulfate. Limiting uptake of either of these molecules will inhibit growth *in silico*. Although the uptake fluxes are small compared to the uptake of the carbon sources and oxygen, both are required for growth of *M. tb*. Consequently, the sulfate and phosphate transporters may serve as bacteriostatic and possibly bacteriocidal drug targets. A robustness analysis (see Methods) was carried for biomass production by *iNJ*661 for phosphate and sulfate [see Additional file 7]. This was compared to a robustness analysis performed for the *in silico E. coli *strain *iJR*904 [24] on aerobic glucose conditions [see Additional file 7]. The sensitivity of the respective biomass production rates to the uptake fluxes are given by the slopes of the plots for each organism. The sensitivity to the phosphate uptake rate for *iNJ*661 and *iJR*904 are both approximately 1. However, the slope for sulfate uptake in *iNJ*661 is approximately four times that of *iJR*904 (about 16 for *iNJ*661 compared to about 4 for *iJR*904). This difference reflects the relatively larger amount of sulfur as a percent of total biomass in *M. tb *compared to *E. coli*, which is consistent with knowledge of *M. tb*'s composition [25]. Collectively, the phase planes and robustness plots suggest that *M. tb *may be much more sensitive to sulfate depletion than *E. coli*.

**Figure 3:**
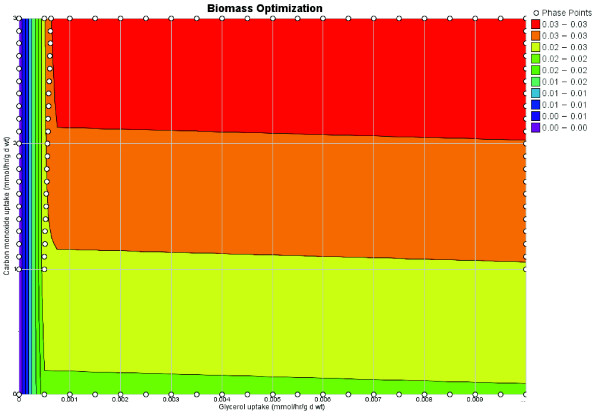
Biomass optimization on Middlebrook media without glucose. Carbon monoxide uptake can affect the growth at very low glycerol uptake rates via Carbon Monoxide Dehydrogenase, which generate protons through the oxidation of carbon dioxide.

A remarkable property of *M. tb *is its ability to grow in culture with carbon monoxide (CO) serving as the sole carbon source [26,27]. Unlike other mycobacteria that can grow with CO as the sole carbon source, *M. tb *lacks ribulose-bisphosphate carboxylase (RuBisCO), an enzyme found in plants enabling fixation of carbon dioxide. Although it was argued that a reductive citric acid cycle may enable the fixation of carbon dioxide into by *M. tb *[27,28], experimentally it was observed that citrate did not affect *M. tb *growth on CO [27]. We did not expect *iNJ*661 to grow on CO, since the only experimentally characterized CO associated metabolism in *M. tb *was CO dehydrogenase (CODH). The uptake of glycerol versus the uptake of CO while optimizing for growth can be seen in Figure 3. CO uptake affects the biomass optimization only during minimal uptake of glycerol, over a slight range. The contribution being made in this region is the donation of protons (through the oxidation of CO to CO_2_). Looking further into this issue, we tested the hypothesis that RuBisCO would enable growth on CO (even though *M. tb *is not known to have it). We added RuBisCO in addition ribose 1-phosphokinase and ribose 1,5-bisphosphate isomerase (to complete the pathway) and optimized for growth on a modified Middlebrook 7H9 media (glycerol and glucose were removed and CO was added). *iNJ*661 was unable to grow under these conditions, despite conferring the ability to fix CO_2 _with ribose sugars. This observation in conjunction with Figure 2a suggests that one possibility for how *M. tb *is able to grow using CO as the carbon source is that it somehow is able to fix CO into a glycolytic intermediate that does not appear to occur through a RuBisCO pathway or a reductive citrate cycle.

### A context for content

**Figure 4:**
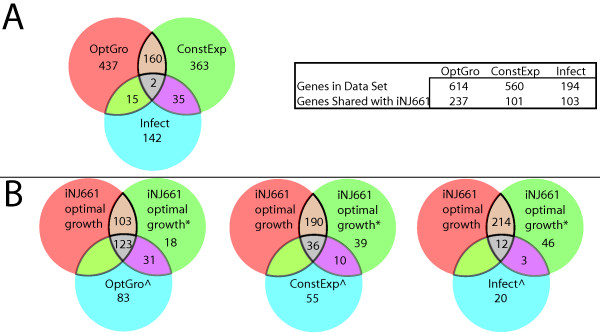
Summary of experimental datasets and their overlap with *iNJ*661 and the *iNJ*661 gene deletion studies. OptGro refers to the dataset described in [23], ConstExp refers to the dataset described in [29], and Infect describes the dataset in [30]. ^ denotes the subset of genes from the experimental dataset contained in *iNJ*661. *iNJ*661 represents the 661 genes in the reconstruction. *iNJ*661 optimal growth represents the subset of genes required for optimal growth with the original objective function. *iNJ*661 optimal growth* represents the subset of genes required for optimal growth with the objective function expanded to include vitamins and cofactors. *iNJ*661 alternative objective represents the set of genes required for optimal growth using the objective function constructed based on the ConstExp dataset. Panel A: The Venn diagram shows the overlap between the different experimental gene expression datasets. The accompanying chart summarizes the total number of genes in each dataset and how many of those genes are found in the *iNJ661 *reconstruction. Panel B: Series of Venn diagrams summarizing the results for the gene deletion studies carried out of *iNJ*661 compared to the experimental gene expression datasets.

This BiGG reconstruction can be used as a 'context for content' in analyzing large biological datasets. Multiple investigators have employed high-throughput data analysis methods to characterize *M. tb *in different environmental conditions, Sassetti et al [23] used transposon site hybridization (TraSH) to identify the genes needed for optimal growth of *M. tb in vitro *(this will be referred to as the OptGro dataset throughout the rest of the text), Gao et al [29] identified a set of genes that are consistently expressed under different growth conditions in liquid culture (this will be referred to as the ConstExp dataset throughout the rest of the text), and Sassetti in 2003 investigated the set of genes needed for *M. tb *survival in vivo during infection in mice [30] (will be referred to as the Infect dataset). The degree of overlap between the three datasets can be seen in Figure 4A (only 2 genes are shared by all three sets) highlight the variability of gene expression across the different experimental datasets. Although significant differences between *in vivo *and *in vitro *patterns of gene expression may be expected, the OptGro and ConstExp datasets were both done *in vitro*. The heterogeneity among the datasets (all three experimental datasets share only two loci) suggests that defining an objective function based on *in vitro *data, may not necessarily translate to useful results *in vivo *(it may constrain the solution space from the wrong directions).

We evaluated these three datasets for *M. tb *and compared them to gene deletion analyses for *iNJ*661, in which each gene was individually deleted and growth was optimized on Middlebrook media. Any growth rate that fell short of the maximum wild type growth was considered 'sub-optimal'. Table 5a summarizes the 114 false negative (*iNJ*661 gene non-essentiality, but *in vitro *gene essentiality; 50%) results between *iNJ*661 and the OptGro dataset (for the full results [see Additional file 4]). The inconsistencies were considered individually and classified into four categories, Not in Objective Function (NOF), Alternate Route (AR), Alternative Locus (AL), and Not Essential (NE); these classifications are not mutually exclusive and were defined in order to navigate and interpret with greater ease. A gene was placed in the NOF category if it was identified to be in a biosynthetic pathway (based on network connectivity) for a particular metabolite, such as a vitamin or co-factor. Genes would be categorized as AR or AL if there was an alternative biochemical route or an alternative gene annotation that could carry out that reaction in *iNJ*661, respectively, but not in *M. tb*. Potential AR or AL classifications reflect one of the potential errors due to sequence based annotations, so further biochemical characterization of the gene products would help resolve these cases. Genes that did not fall in these categories were classified as NE. Many of these loci were associated with fluxes in which the flux variability was uniformly zero (maximum and minimum value).

**Table 5a:** Classification of False Negative results by subdivision into four overlapping categories for the gene deletion study in *iNJ*661 compared to the OptGro dataset. Each row and column lists the number of those genes in the respectively classes. NOF: Not in Objective Function, AR: Alternate Route, AL: Alternative Locus, NE: Not essential.

**Classification of False Negative results**				
	**AL**	**AR**	**NE**	**NOF**

**AL**	26	0	5	2
**AR**	0	7	6	1
**NE**	5	6	24	0
**NOF**	2	1	0	43

Since quantitative data was not available for vitamins and cofactors, they were not included in the original definition of the objective function (Table 2). However, after the deletion analysis, the majority of the false negative predictions in the NE category by *iNJ*661 were reactions associated with the biosynthesis of vitamins and cofactors, such as dihydrofolate and tetrahydrofolate. 16 such metabolites were added [see Additional file 1] to the biomass function with coefficients of 0.000001 (small enough to have negligible effects on quantitative growth, but still required for growth). The results for the genes required for optimal growth using the initial biomass function and the expanded compared to OptGrow are displayed in the first Venn diagram of Figure 4B. Although only 16 metabolites were added, the deletion study resulted in 31 more gene loci that matched the OptGro data. This only slightly increased the deletion prediction from 54% to 56%. The reason for the modest increase is that there were also 18 additional false positive (*iNJ*661 gene essentiality, but *in vitro *gene non-essentiality) results (from 46% to 44% for the original and expanded objective functions, respectively).

Genes categorized as AL or AR suggest that there was an error in the sequence based annotation, there are regulatory control loops that we are not aware of, or there was a false positive result from the experimental data (TraSH is often used as a screening tool). Sassetti et al [23] dicussed one of these cases involving the Rv0505c and Rv3042c loci (serB and serB2 respectively). Both have been assigned the same function based on annotation, however serB2 was found to be essential while serB was not. There may be many possibilities why serB cannot rescue mutations/deletions of serB2, however the detection of such cases imply that having duplicate genes many not confer the degree or robustness that is often assumed to accompany an organism. The cases with alternative loci highlight areas where further experimental investigation may provide insight into the growth capabilities of *M. tb*. The AL cases are enumerated in Table 5b. These loci are worth further experimental investigation to confirm the annotation and to determine the different conditions which may induce or inhibit expression. The false negative prediction for the sulfate transporter (Rv2397c, Rv2398c, Rv2399c, and Rv2400c) is an interesting case to consider, in part because it validates the observations in the phase plane discussed in Figure 2b. Additionally, it identifies an area in which a partially characterized annotation causes erroneous results. The four aforementioned loci form a protein complex in the model in order to catalyze the transport reaction. However, since there was an additional annotation identifying Rv1739c as a sulfate transporter [31], an association was made between Rv1739c in addition to the existing protein complex (Rv2397c + Rv2398c + Rv2399c + Rv2400c). So, although the sulfate transporter is essential for *in silico *growth of *iNJ*661, the lack of a biochemically characterized gene protein relationship can result in false predictions.

**Table 5b:** Detailed listing of the AL results for the False Negative results for *iNJ*661. The first column lists a locus needed for optimal growth according to the OptGro dataset. The second column lists the reactions involved. The third column lists the possible alternative loci in *iNJ*661 (commas indicate isozymes, addition symbols indicate formation of protein complexes).

**Alternative Loci in False Negative results**		
**OptGro**	**Reaction**	**Other loci in iNJ661**

Rv0337c	ASPTA	Rv2565
Rv0462	PDHc, PDH, PDHa	Rv0843, Rv2497c+Rv2496c
Rv0808	GLUPRT	Rv1602
Rv0824c	DESAT18	Rv1094
Rv1122	GND	Rv1844c
Rv1600	HSTPT	Rv3772, Rv2231c
Rv1602	GLUPRT	Rv0808
Rv1609	ANS	Rv2859c
Rv2182c	AGPAT160190, AGPAT160, AGPAT190	Rv2483c
Rv2215	PDH, AKGDb	Rv2241, Rv0462, Rv0843
Rv2231c	HSTPT	Rv1600, Rv3772
Rv2397c	SULabc, TSULabc	Rv1739c
Rv2398c	SULabc, TSULabc	Rv1739c
Rv2399c	SULabc, TSULabc	Rv1739c
Rv2400c	SULabc, TSULabc	Rv1739c
Rv2682c	DXPS	Rv3379c
Rv2746c	PGSA190, PGSA160, PGSA160190	Rv1822
Rv2996c	PGCD	Rv0728c
Rv3003c	ACLS	Rv3509c+Rv3002c, Rv1820+Rv3002c, Rv3509c+Rv3002c, Rv3002c+Rv3470c
Rv3042c	PSP_L	Rv0505c
Rv3257c	PMANM, AIRCr	Rv3275c+Rv3276c
Rv3275c	AIRCr	Rv3257c+Rv3276c
Rv3281	ACCOACr	Rv3280+Rv0904c
Rv3441c	PMANM, ACGAMPM	Rv3308, Rv3257c
Rv3634c	UDPG4E	Rv0536, Rv0501
Rv0112	GMAND	Rv1511
Rv2247	PPCOAC	Rv2502c+Rv0973c, Rv3285+Rv0974c, Rv0973c+Rv0974c,
Rv0973c	PPCOAC	Rv2502c+Rv0973c, Rv3285+Rv0974c, Rv0973c+Rv0974c, Rv2501c+Rv2247
Rv3285	PPCOAC	Rv2502c+Rv0973c, Rv0973c+Rv0974c, Rv2501c+Rv2247
Rv2967c	PC	Rv2976c
Rv3464	TDPGDH	Rv3468c, Rv3784
Rv2754c	TMDS	Rv2764c
Rv3713	ADCYRS	Rv2848c

The 103 false positive cases likely reflect incomplete knowledge about the network (alternative pathways exist) or an alternative objective function [see Additional file 4]. Approximately 20 cases involve amino acid pathways, since amino acids would presumably be required for any kind of growth in lab conditions, these cases likely suggest alternative pathways or enzymes are present in *M. tb *that are not in iNJ661. Almost half of these cases (46) involve fatty acid, membrane, or peptidoglycan metabolism, since the fatty acid composition in *M. tb *can change significantly over time *in vitro *[9], many of these false predictions are likely to be due to changes in the biomass composition (*in silico *simulations have fixed biomass compositions). These cases highlight areas in which experimental studies documenting the changes in composition and growth rates may yield interesting results. Five of the cases involve loci associated with the transport of phosphate, ammonium, and a sugar transporter. These cases likely involve as of yet unidentified alternative transporters in *M. tb*. Another case involves glycerol kinase (Rv3696c, GlpK, GLYK) which was one of the fluxes with no flexibility (see ***M. tb *growth *in silico***), since *M. tb in vitro *requires glycerol supplementation for growth [20]. However, since glycerol kinase does not appear to be required even for sub-optimal growth as implied by the OptGro dataset, there must be other reactions involving the direct metabolism of glycerol that have not yet been identified for *M. tb*. The ability to grow using CO as the sole carbon source and maintain optimal growth even without glycerol kinase activity imply unique and unidentified aspects to central metabolism in the *M. tb *network. Further experimental investigations may provide clues towards controlling the growth of *M. tb *(increasing the growth rate in culture and inhibiting growth *in vivo*).

Out of the 194 genes identified in the Infect dataset, only 17 were shared with the OptGro dataset and 37 were shared with the ConstExp dataset (Figure 4A). From these three sets of experimental datasets, OptGro and ConstExp had the greatest number of genes in common, 162. However this is still only about 27% and 29% of the genes in each dataset, respectively. The large variability between all of these datasets highlights the point that further development of Flux Balance Analysis based approaches will require the definition alternative objective functions to biomass optimization.

### Functional assessment independent of objective functions

Biomass functions can help improve predictions under well-defined conditions by constraining the steady state solution space. However, different growth conditions can appreciably alter the composition and the 'objectives' of bacteria, as reflect by Figure 4A and the discussion above. Significant changes in fatty acid composition have been observed even while growing *M. tb *in culture over the span of weeks [12]. These changes would be reflected as changes in the composition and the coefficients of the biomass function, consequently altering the predictions made by the model. Not all COBRA methods require the definition of objective functions [32] and identifying groups of reactions that operate together can help simplify a network and provide insight into its functionality [33]. Correlated reaction sets have been calculated using sampling [34] and flux coupling [35] and implications for classifying diseases and identifying pathway specific drug targets have been discussed [36]. Applying the same systems view of metabolic networks to pathogens such as *M. tb *causes one to adopt the view that single enzyme drug targets actually knock out complete pathways. As a result, terminating the activity of any other enzyme in that pathway should have the same effect. Similar to the correlated reaction sets calculated by sampling or flux coupling, HCR sets are defined by mass balance constraints. The definition of HCRs is stricter in the sense that it is based on metabolites with one-to-one connectivity. However in contrast to the other approaches, it is independent of the exchange reactions with the environment and any requirements for demand functions. Consequently, the sets can be calculated directly from the stoichiometric matrix. There is a significant degree of overlap between HCRs and the Enzyme Subsets (ES) described by Pfeiffer et al [37]. Since ESs are not constrained by 1:1 connectivity, in principle they may include additional reactions. On the same token, when the same reaction is carried out by multiple co-factors and the reactions are reversible, which is not uncommon in metabolic networks, ESs may add reactions which form loops (similar to Type III Extreme Pathways [38]). Additionally, intermediate steps in the calculation of HCRs allow the consideration of inclusion or removal of potentially reversible reactions.

**Figure 5:**
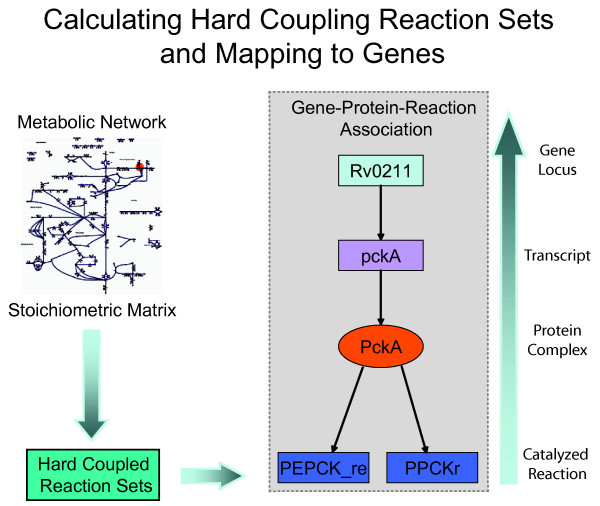
The stoichiometric matrix is created from the metabolic network. The HCR (Hard Coupled Reaction) sets are calculated directly from the stoichiometric matrix and mapped back to the gene loci. Gene-Protein-Reaction relationships: top box is the gene, the next box is the peptide, the oval represents the functional protein, and the bottom boxes are the reactions catalyzed by the protein.

147 HCR sets (1:1 only, additional reversible reactions, 0:2/2:0, were not included, see Methods) were calculated for the network, the average size of each set was 2.93, with median and modes both equal to 2. The largest set, HCR 40, consisted of 13 reactions involved in Cofactor Metabolism and Porphyrin Metabolism. The summary of how the HCRs are split among the 35 subsystems in the network are outlined in Table 6. Using the Gene-Protein-Reaction relationships which form the foundation of the reconstruction, we mapped the HCRs back to the gene loci (Figure 5). The complete HCR set mapped to 124 loci and 61 HCRs in the OptGro dataset, to 46 loci and 34 HCRs in the ConstExp dataset, and 21 loci and 18 HCRs in the Infect dataset [see Additional file 2]. The OptGro and ConstExp shared 8 HCR sets (HCR 2, 3, 11, 12, 43, 57, 69, and 122). The intersection of these datasets identifies a subset of reactions that are consistently expressed under different conditions and also required for optimal growth *in vitro*. These HCR sets may reflect underlying core sets of reactions vital to growth and survival *in vitro*.

**Table 6:** HCR sets mapped to the subsystems in the network along with the size of each subsystem.

**HCR mapping to subsystems**		
**Metabolic Subsystem**	**HCR Sets in each Subsystem**	**Number of HCRs**

Alanine and Aspartate Metabolism	47,81,99,126	4
Arginine and Proline Metabolism	126	1
Biotin Metabolism	33	1
Citric Acid Cycle	98	1
Cofactor Metabolism	7,24,25,26,30,40,56,77,118,123,129	11
Cysteine Metabolism	38	1
Fatty Acid Metabolism	1,43,57,78,86,90,93,94,95,96,97,103,108,110,114,115,116,128,132	19
Folate Metabolism	9,22,28,31,44	5
Glutamate Metabolism	21,27,29,67,76	5
Glycine Serine Threonine Metabolism	19,24,45,111,124	5
Glycolysis	41,42	2
Glyoxylate Metabolism	104	1
Histidine Metabolism	10,70	2
Lysine Metabolism	2,8	2
Membrane Metabolism	23,29,34,35,36,43,46,61,64,65,66,74,79,97,103,106,107,108,109,110,112,113,116,117,130,131,138	27
Methionine Metabolism	27,28,45,91,92	5
Nucleotide Sugar Metabolism	55,59,63,69,75,83,140	7
Other Amino Acid Metabolism	29,48,82,91,92,125,127	7
Pantothenate and CoA Metabolism	12,67,68,81	4
Pentose Phosphate Pathway	32,121	2
Peptidoglycan Metabolism	43,133,134,136,137,100	6
Phenylalanine Tyrosine Tryptophan Metabolism	10,11,20,85,105	5
Polyprenyl Metabolism	16,60,62,77	4
Porphyrin Metabolism	25,30,40,51,52,53	6
Purine Metabolism	5,17,27,49,58,63,73,140	8
Pyrimidine Metabolism	55,59,83,98	4
Pyruvate Metabolism	82	1
Redox Metabolism	39,50,71,72,76,80,100,102	8
Riboflavin Metabolism	6,7,26,30,56	5
Sugar Metabolism	15,42,74,75,87,89,99,119,120,135	10
Thiamine Metabolism	15	1
Transport	39,48,54,71,82,84,87,88,89,101,102,106,110,124,127,139,141,142,143,144,145,146,147	23
Ubiquinone Metabolism	13,122,125	3
Urea Cycle	27,37	2
Valine Leucine Isoleucine Metabolism	3,4,14,18,84,88,139	7

**Figure 6:**
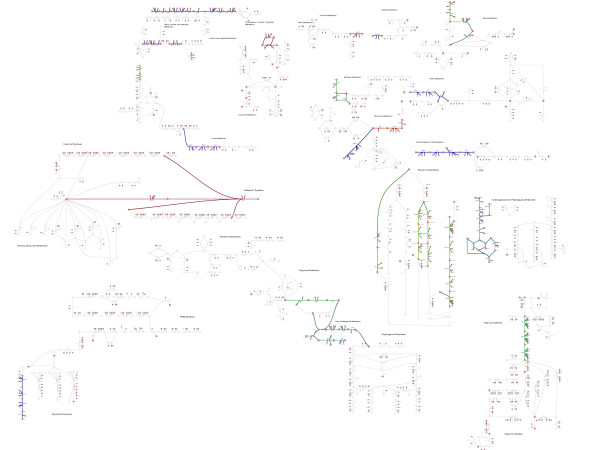
A partial metabolic map of *iNJ*661 with the 25 drug target HCR sets. Each reaction is numbered and color coded according to the HCR which it belongs to. The number of the HCR sets matches those in the Additional files. Only pathways or parts of pathways which included members of the 25 HCRs are depicted.

Many of the tuberculosis treatment drugs have metabolic targets [1] and a use of this metabolic based reconstruction can be to assist in identifying new and alternative chemotherapy targets for tuberculosis. Mdluli and Spigelman recently discussed the drug targets in *M. tb *reasonably comprehensively [39]. We used the list (absent the DNA synthesis and Regulatory Protein targets) and mapped them to the 147 HCR sets as seen in Table 7 [see Additional file 3 for the full set]. The final column lists the number of different reactions in the HCR. Other reactions that are shared within a particular HCR which contains a known drug target, have the potential to be alternative but metabolically equivalent targets. The drug targets mapped to 25 of the HCR sets. These sets are depicted on a reduced metabolic map of *iNJ*661 (Figure 6). An image of the HCR sets mapped to the entire metabolic network will be available on our website [40]. These 25 HCR sets contain all of the 8 HCR sets identified by the overlap between the OptGro and ConstExp data.

**Table 7:** Mapping the HCR sets to the drug targets described by Mdluli and Spigelman [39]. The first column includes the protein names (adopted from Mdluli and Spigelman), the second column lists the corresponding gene locus, the third is the HCR set number and the fourth lists how many reactions are in the HCR set. Only those drug targets mapped to an HCR are shown. [See Additional file 3] for the full set of HCR sets and the individual reactions they are comprised of; the HCR sets mapped to the above drug targets are highlighted in these data.

**HCRs mapped to *Mtb *drug targets**			
**Drug Target**	**Gene Locus**	**HCR**	**HCR size**

EmbA	Rv3794	43	10
EmbB	Rv3795	43	10
EmbC	Rv3793	43	10
AftA	Rv3792	43	10
decaprenyl phosphoryltransferase	Rv3806c	62	3
udp galatofuraosyltransferase	Rv3808c	43	10
dTDP-deoxy-hexulose reductase	Rv3809c	43	10
RmlA	Rv0334	69	4
RmlB	Rv3464	69	4
RmlC	Rv3465	69	4
RmbD	Rv3266c	69	4
InhA	Rv1484	57	3
MabA	Rv1483	90	5
KasA	Rv2245	90	5
KasB	Rv2246	90	5
MmaA4	Rv0642c	93	4
Pks13	Rv3800c	86	4
FadD32	Rv3801c	86	4
AccD5	Rv3280	78	2
LeuD	Rv2987c	14	2
TrpD	Rv2192c	10	4
DapB	Rv2773c	2	3
AroA	Rv3227	20	3
AroC	Rv2540c	20	3
AroE	Rv2537c	11	4
AroG	Rv2178c	11	4
AroQ	Rv2537c	11	4
ilvG (acetolactate synthase)	Rv1820	3	3
ilvX (acetolactate synthase)	Rv3509c	3	3
ilvN (acetolactate synthase)	Rv3002c	3	3
ilvB (acetolactate synthase)	Rv3003c	3	3
ilvB2 (acetolactate synthase)	Rv3470c	3	3
branchd chain aminotransferase	Rv2210c	84	2
dihyropteroate synthase	Rv3608c	9	3
dihyropteroate synthase	Rv1207	9	3
PanC	Rv3602c	12	4
riboflavin synthase	Rv1416	56	3
riboflavin synthase	Rv1412	56	3
sulfotransferase	Rv1373	131	2
MshA	Rv0486	80	2
MshB	Rv1170	80	2
MshC	Rv2130c	50	2
MshD	Rv0819	50	2
IspD	Rv3582c	16	5
IspE	Rv1011	16	5
IspF	Rv3581c	16	5
MenA	Rv0534c	13	2
MenB	Rv0548c	122	3
MenD	Rv0555	125	2
MenE	Rv0542c	122	3

Last year Raman et al [41] constructed and analyzed a model of the mycolic acid synthesis pathways and proposed additional targets in fatty acid metabolism. Accounting for the entire metabolic network further builds upon this by enabling a more rigorous evaluation of the global growth capabilities of the network and the identification of potential drug targets. The genome-scale model presented here includes the extensive fatty acid metabolism pathways of *M. tb*, in addition to rest of the metabolic network. With this full network, we then sought to extend the identification of potential drug targets by taking advantage of the underlying principles of reconstructing metabolic networks to calculate HCR sets.

Interpreting large, complex networks in a functionally meaningful manner is enabled by adopting a hierarchical view of the network, where one identifies groups of reactions that respond in unison to changes in media conditions. Groups of reactions strictly bound together based on mass conservation and stoichiometry constraints can lead to the definition of hierarchical sets of reactions [33,34]. This principle was applied to causal SNP associated diseases in the human mitochondria, and it was found that reactions in the same co-set had similar disease phenotypes [36]. We applied this same concept to *iNJ*661, but rather than looking for disease phenotypes, we sought to identify alternative drug targets.

## Conclusion

Over time and with iterative improvements, metabolic network reconstructions have achieved the stage of hypothesis generation and model-driven biological discovery in systems biology [42]. We present an *in silico *strain of *M. tb, iNJ*661, with the anticipation that it will be received by the community in a similar vein and to assist in the discovery process of this remarkable yet devastating pathogen.

A large amount of material has been presented in this manuscript, beginning with the presentation of a genome-scale reconstruction and model of *M. tb*, *iNJ*661, followed by experimental validation and use of the model for integration and analysis of multiple experimental datasets. We summarize some of the main points that highlight areas in which further experimental research may be fruitful.

### Growth studies

• *iNJ*661 can grow at rates consistent with experimental data in varying media conditions. Further measurements of the biomass composition of *M. tb *in well-defined situations are likely to improve the *in silico *predictions under those conditions.

• Analysis of the capabilities under maximal growth conditions can help identify critical nutrient targets for killing *M. tb *(such as the sulfate transporter) or for optimizing growth (in the lab).

• The pathway enabling growth of *M. tb *using CO as the carbon source is still unclear. The computational studies suggest that there must be an alternative pathway (to reductive citrate cycle and RuBisCO) which enables entry of CO_2 _into central metabolism.

• The seemingly paradoxical need for glucose supplementation in Middlebrook media but non-essentiality of directly related reactions involving the metabolism of glucose-6-phosphate for *in vitro *growth and the almost diametrically opposed results for *in silico *growth, suggest that there may be additional pathways involving glucose metabolism and potentially non-metabolic functions related to the glucose requirement.

### Comparison with and analysis of large datasets

• The definition of the biomass function will largely dictate the results of optimal growth/gene deletion experiments, consequently, positive results (in agreement with the experiments) are not as interesting as the false negative (or false positive results). The false negative results may be due to an error in the annotation of the gene or an underlying physiological mechanism that we are not aware of. In either case, investigating the particular genes experimentally may help clarify the issue.

• The model can be used as a Context for Biological Content and provides a consistent, coherent framework to analyze various datasets focusing on metabolism.

### 'Unbiased' analysis

Unbiased analysis methods, methods that calculate properties of the network independent of objective functions, can provide interesting and valuable insights into network capabilities [32]. The definition of HCR sets, in a similar vein to correlated reaction sets from sampling and flux-coupling, can confer a hierarchical structure to metabolic networks, and may assist in the identification of alternative drug targets. Using these models in conjunction with experimental data can assist in screening and identifying new and alternative drug targets.

Taken together, this study adds another high resolution reconstruction of microbial metabolism. There now exist a growing number of such reconstructions that have been particularly useful for basic studies of network properties and for bioprocessing applications [42-44]. As the number of reconstructions of human pathogens grows, we should be able to expand their uses towards improved understanding pathogenic mechamisms, and design of interventions and treatment. Large-scale rigorous experimental validation studies should now be performed to further these goals.

## Methods

Reconstruction and model development of the metabolic network has been described previously [7,8]. The reconstruction contents will be made available on our website [40]  in Excel [see Additional file 5] and SBML formats.

### Reconstruction

Figure 1 provides a brief summary of the reconstruction and modeling building process. The sequence based genome annotation of Mycobacterium tuberculosis H37Rv was downloaded from TIGR [45] and served as the framework of the model. Charge and elementally balanced reactions were added individually based on this annotation, legacy data when available, and updates made in the Tuberculist database [31]. KEGG [46] and the SEED [47] were used as ancillary tools on occasion. Following the initial reconstruction, the gaps were evaluated individually by searching for direct evidence in the literature for their metabolism. Due to the relative sparsity of literature on aspects of central and cofactor metabolism in *M. tuberculosis*, many of these gaps could not be filled. Legacy data in the form of primary articles, review articles, and textbooks were employed in addition to the database resources [see Additional file 6] during the reconstruction and model building phases. A comprehensive map of *iNJ*661's metabolic network was visualized by creating a map of the network organized by lumped subsystems of metabolism [see Additional files 8, 9, 10, 11, 12, 13, 14, 15]. After the debugging process, when the cell could grow (i.e. produce biomass) on different media, the remaining intracellular gaps were evaluated by searching the literature for evidence of metabolic reactions involving that particular metabolite. If no evidence of transport or biochemical transformations of the metabolite in *M. tb *was found, no additional reactions or transporters were added.

### Model formulation

The stoichiometric matrix, S, was constructed based on the reactions described in the reconstruction. The biomass function was defined using available legacy data [see Additional file 1]. Flux Balance Analysis (FBA) simulations were employed during the debugging process towards developing a functional model.

### Flux Balance Analysis (FBA)

The metabolic network is represented mathematically by the stoichiometric matrix, **m **rows by **n **columns, where there are **m **metabolites and n reactions in the network. Mass conservation dictates

dxdt=S•v
 MathType@MTEF@5@5@+=feaafiart1ev1aaatCvAUfKttLearuWrP9MDH5MBPbIqV92AaeXatLxBI9gBaebbnrfifHhDYfgasaacH8akY=wiFfYdH8Gipec8Eeeu0xXdbba9frFj0=OqFfea0dXdd9vqai=hGuQ8kuc9pgc9s8qqaq=dirpe0xb9q8qiLsFr0=vr0=vr0dc8meaabaqaciaacaGaaeqabaqabeGadaaakeaadaWcaaqaaiabdsgaKjabdIha4bqaaiabdsgaKjabdsha0baacqGH9aqpcqWGtbWucqGHIaYTcqWG2bGDaaa@3777@

which simplifies to

*S*·*v *= 0

at the steady state. Further constraints can be placed on the system based on thermodynamics (reaction directionality) and limits on substrate uptake rates

*α *≤ *v *≤ *β*

The oxygen uptake rate measured from *the Mycobacteria Bovis *BCG strain of 25 μL/hr/mg dry weight (0.98 mmol O_2_/hr/g dry weight) [48] was used. The small molecule carbon sources (glucose, glycerol, etc.) were limited to an uptake rate of 1 mmol/hr/g dry weight and all other allowable substrates were unconstrained. The different *in silico *culture media are listed in Table 3. Deviations from the standard media include: the addition of minute amounts of ferric iron in Youmans media. The oxygen uptake rate was the growth constraining flux in all of the different media.

### Flux Variability Analysis (FVA)

Constraining the biomass objective function, c, to the optimal value calculated in FBA,

max(*c*^*T*^·*v*)

every flux in the network is then minimized and maximized. The different between the maximum and minimum for each flux defines the flux variability for that reaction.

### Robustness Analysis

Robustness plots are performed by varying a particular flux through a pre-defined range and recalculating the objective function. The slope of the curve describes the sensitivity of the objective function on that particular flux (over the specified range of values).

### Hard-Coupled Reaction (HCR) Sets

Denoting the binary form of the stoichiometric matrix, S^
 MathType@MTEF@5@5@+=feaafiart1ev1aaatCvAUfKttLearuWrP9MDH5MBPbIqV92AaeXatLxBI9gBaebbnrfifHhDYfgasaacH8akY=wiFfYdH8Gipec8Eeeu0xXdbba9frFj0=OqFfea0dXdd9vqai=hGuQ8kuc9pgc9s8qqaq=dirpe0xb9q8qiLsFr0=vr0=vr0dc8meaabaqaciaacaGaaeqabaqabeGadaaakeaacuqGtbWugaqcaaaa@2DE9@, the input/reactant part of the stoichiometric matrix, S_-_, and the output/product part of the stoichiometric matrix, S_+_, then it is transparent that,

S=S^−+S^+
 MathType@MTEF@5@5@+=feaafiart1ev1aaatCvAUfKttLearuWrP9MDH5MBPbIqV92AaeXatLxBI9gBaebbnrfifHhDYfgasaacH8akY=wiFfYdH8Gipec8Eeeu0xXdbba9frFj0=OqFfea0dXdd9vqai=hGuQ8kuc9pgc9s8qqaq=dirpe0xb9q8qiLsFr0=vr0=vr0dc8meaabaqaciaacaGaaeqabaqabeGadaaakeaacqWGtbWucqGH9aqpcuWGtbWugaqcamaaBaaaleaacqGHsislaeqaaOGaey4kaSIafm4uamLbaKaadaWgaaWcbaGaey4kaScabeaaaaa@3472@

The input and output connectivities of each metabolite can be calculated for S^
 MathType@MTEF@5@5@+=feaafiart1ev1aaatCvAUfKttLearuWrP9MDH5MBPbIqV92AaeXatLxBI9gBaebbnrfifHhDYfgasaacH8akY=wiFfYdH8Gipec8Eeeu0xXdbba9frFj0=OqFfea0dXdd9vqai=hGuQ8kuc9pgc9s8qqaq=dirpe0xb9q8qiLsFr0=vr0=vr0dc8meaabaqaciaacaGaaeqabaqabeGadaaakeaacuqGtbWugaqcaaaa@2DE9@_- _and S^
 MathType@MTEF@5@5@+=feaafiart1ev1aaatCvAUfKttLearuWrP9MDH5MBPbIqV92AaeXatLxBI9gBaebbnrfifHhDYfgasaacH8akY=wiFfYdH8Gipec8Eeeu0xXdbba9frFj0=OqFfea0dXdd9vqai=hGuQ8kuc9pgc9s8qqaq=dirpe0xb9q8qiLsFr0=vr0=vr0dc8meaabaqaciaacaGaaeqabaqabeGadaaakeaacuqGtbWugaqcaaaa@2DE9@_+_, respectively. The input:output ratio for each metabolite can be determined and all metabolites with 1:1 connectivity will be hard-coupled by the network. Metabolites with 0:2 or 2:0 connectivity reflect metabolites that are hard-coupled in the event that the corresponding reaction is reversible. Metabolites with 0:1 or 1:0 connectivity are either source/sink transporters or blocked reactions.

A general algorithm for calculating hard coupled reaction sets.

1. Identify 1:1, 0:2, and 2:0 metabolites

2. Define HCRs

a. Identify the sets of reactions corresponding to the metabolites, denote the entire set as HCR_0_

b. Join any HCR_0 _subset that share 1 or more reactions, denote the entire set as HCR_1_

c. i ← 1

3. while (HCR_i-1 _≠ HCR_i_)

a. Join any HCR_i _subset that share 1 or more reactions

b. i ← i+1

Since 0:2 or 2:0 reactions may be irreversible reactions, an intermediate processing step may be needed to verify that there is biological evidence supporting the catalysis of the reaction in the reverse direction. If one does not wish to consider these potential effects and assume that all of the reactions are reversible then step 1 can be simplified by finding all of the 2 metabolites for S^
 MathType@MTEF@5@5@+=feaafiart1ev1aaatCvAUfKttLearuWrP9MDH5MBPbIqV92AaeXatLxBI9gBaebbnrfifHhDYfgasaacH8akY=wiFfYdH8Gipec8Eeeu0xXdbba9frFj0=OqFfea0dXdd9vqai=hGuQ8kuc9pgc9s8qqaq=dirpe0xb9q8qiLsFr0=vr0=vr0dc8meaabaqaciaacaGaaeqabaqabeGadaaakeaacuqGtbWugaqcaaaa@2DE9@. Biomass functions were removed from the stoichiometric matrix before calculating the HCR sets. The HCRs calculated for *iNJ*661 had 94 0:2/2:0 reactions. Of these, 24 overlapped with the core set of 147 1:1 HCRs. Since the set of 0:2/2:0 reactions did not have additional gene associations (that weren't already in the 1:1 set) and since they had many irreversible reactions throughout, the 1:1 set and the 0:2/2:0 set were not combined (step 1 in the above algorithm outline would involve only identifying the 1:1 metabolites).

The reconstruction process, the metabolic maps, and the majority of the calculations were performed using Simpheny™ v1.11 software (Genomatica, Inc.). HCR Sets were calculated with Mathematica^© ^v4.2 (Wolfram Research, Inc.).

## Authors' contributions

NJ carried out the reconstruction, performed the analyses, and drafted the manuscript. BØP conceived the study and helped revise the manuscript. Both authors approved the content of the final manuscript.

## Supplementary Material

Additional File 1 Molecular composition of biomass functions. A detailed list of the biomass constituents and their respective coefficients in the biomass function in addition to the references from which this information was found. Click here for file

Additional File 7 Robustness plots for phosphate and sulfate for *iNJ*661 and *iJR*904. Plotting the biomass objective function versus phosphate or sulfate uptake rates for *in silico *strains of *M. tb *and *E. coli*. Click here for file

Additional File 4 Comprehensive list of false negatives between OptGro data set and *iNJ*661 lethal gene deletions. A catalog of the false negative results produced by *iNJ*661 when compared to the OptGro data set. Each of false negative results is categorized into at least one of four different categories. Specific loci are listed in cases with suggested alternative loci or alternative paths. Click here for file

Additional File 2 HCRs mapped to experimental data sets. The list of HCRs that mapped to the three experimental data, based on gene loci. Click here for file

Additional File 6 Explicit list of references used in the reconstruction process. The list of references used to define particular reactions and GPR relationships. Click here for file

Additional File 8 Amino acid metabolism. A map of the amino acid pathways in *iNJ*661. Click here for file

Additional File 9 Central metabolism. A map of the central metabolic pathways in *iNJ*661. Click here for file

Additional File 10 Fatty acid metabolism. A map of the fatty acid pathways in *iNJ*661. Click here for file

Additional File 11 Membrane metabolism. A map of the membrane metabolism pathways in *iNJ*661. Click here for file

Additional File 12 Nucleotide metabolism. A map of the nucleotide synthesis and degradation pathways in *iNJ*661. Click here for file

Additional File 13 Peptidoglycan metabolism. A map of the peptidoglycan pathways in *iNJ*661. Click here for file

Additional File 14 Transporters. A map of the transport reactions found in *iNJ*661. Click here for file

Additional File 15 Vitamin and cofactor metabolism. A map of the vitamin and cofactor pathways in *iNJ*661. Click here for file

Additional File 3 HCRs mapped to reaction and metabolic subsystems. The complete list of HCRs, highlighting those known to be drug targets in *M. tb*. Click here for file

Additional File 5 Model content for *iNJ*661. A complete list of the reactions (abbreviations and full names), the associated mass and charge balanced reactions, confidence scores, reaction subsystems, and the list of the Gene-Protein-Reaction relationships, including logical AND/OR relationships, for all of the reactions in the model. A complete list of metabolites appearing in *iNJ*661, including abbreviation, full name, formula, and charge at pH 7.2 can be found in the second tab in the file. Click here for file

## References

[B1] Kasper D, Braunwald E, Fauci A, Hauser S, Longo D, Jameson J (2004). Harrison's Principles of Internal Medicine.

[B2] Schneider E, Moore M, Castro KG (2005). Epidemiology of tuberculosis in the United States. Clinics in chest medicine.

[B3] Small PM, Fujiwara PI (2001). Management of tuberculosis in the United States. The New England journal of medicine.

[B4] Sharma SK, Mohan A (2006). Multidrug-resistant tuberculosis: a menace that threatens to destabilize tuberculosis control. Chest.

[B5] Wayne LG, Sohaskey CD (2001). Nonreplicating persistence of mycobacterium tuberculosis. Annual review of microbiology.

[B6] Cole ST, Brosch R, Parkhill J, Garnier T, Churcher C, Harris D, Gordon SV, Eiglmeier K, Gas S, Barry CE (1998). Deciphering the biology of Mycobacterium tuberculosis from the complete genome sequence. Nature.

[B7] Becker SA, Palsson BO (2005). Genome-scale reconstruction of the metabolic network in Staphylococcus aureus N315: an initial draft to the two-dimensional annotation. BMC microbiology [electronic resource].

[B8] Feist AM, Scholten JC, Palsson BO, Brockman FJ, Ideker T (2006). Modeling methanogenesis with a genome-scale metabolic reconstruction of Methanosarcina barkeri. Molecular systems biology [electronic resource].

[B9] Khuller GK, Taneja R, Kaur S, Verma JN (1982). Lipid composition and virulence of Mycobacterium tuberculosis H37Rv. The Australian journal of experimental biology and medical science.

[B10] Nandedkar AK (1983). Comparative study of the lipid composition of particular pathogenic and nonpathogenic species of Mycobacterium. Journal of the National Medical Association.

[B11] Watanabe M, Aoyagi Y, Ridell M, Minnikin DE (2001). Separation and characterization of individual mycolic acids in representative mycobacteria. Microbiology (Reading, England).

[B12] Youmans AS, Youmans GP (1968). Ribonucleic acid, deoxyribonucleic acid, and protein content of cells of different ages of Mycobacterium tuberculosis and the ralationship to immunogenicity. J Bacteriol.

[B13] Garcia-Vallve S, Guzman E, Montero MA, Romeu A (2003). HGT-DB: a database of putative horizontally transferred genes in prokaryotic complete genomes. Nucleic acids research.

[B14] Beste DJ, Peters J, Hooper T, Avignone-Rossa C, Bushell ME, McFadden J (2005). Compiling a molecular inventory for Mycobacterium bovis BCG at two growth rates: evidence for growth rate-mediated regulation of ribosome biosynthesis and lipid metabolism. J Bacteriol.

[B15] Acharya PV, Goldman DS (1970). Chemical composition of the cell wall of the H37Ra strain of Mycobacterium tuberculosis. J Bacteriol.

[B16] Middlebrook G, Cohn ML (1958). Bacteriology of tuberculosis: laboratory methods. American journal of public health.

[B17] Youmans, Karlson (1947).

[B18] James BW, Williams A, Marsh PD (2000). The physiology and pathogenicity of Mycobacterium tuberculosis grown under controlled conditions in a defined medium. Journal of applied microbiology.

[B19] Cox RA (2004). Quantitative relationships for specific growth rates and macromolecular compositions of Mycobacterium tuberculosis, Streptomyces coelicolor A3(2) and *Escherichia coli* B/r: an integrative theoretical approach. Microbiology (Reading, England).

[B20] Primm TP, Andersen SJ, Mizrahi V, Avarbock D, Rubin H, Barry CE (2000). The stringent response of Mycobacterium tuberculosis is required for long-term survival. J Bacteriol.

[B21] Edwards J, Ramakrishna R, Palsson B (2002). Characterizing the metabolic phenotype: a phenotype phase plane analysis. Biotechnol Bioeng.

[B22] Reed J, Palsson B (2004). Genome-scale in silico models of E. coli have multiple equivalent phenotypic states: assessment of correlated reaction subsets that comprise network states. Genome Res.

[B23] Sassetti CM, Boyd DH, Rubin EJ (2003). Genes required for mycobacterial growth defined by high density mutagenesis. Mol Microbiol.

[B24] Reed JL, Vo TD, Schilling CH, Palsson BO (2003). An expanded genome-scale model of Escherichia coli K-12 (iJR904 GSM/GPR). Genome biology.

[B25] Cole ST (2005). Tuberculosis and the tubercle bacillus.

[B26] King GM (2003). Uptake of carbon monoxide and hydrogen at environmentally relevant concentrations by mycobacteria. Appl Environ Microbiol.

[B27] Park SW, Hwang EH, Park H, Kim JA, Heo J, Lee KH, Song T, Kim E, Ro YT, Kim SW (2003). Growth of mycobacteria on carbonmonoxide and methanol. J Bacteriol.

[B28] Srinivasan V, Morowitz HJ (2006). Ancient genes in contemporary persistent microbial pathogens. The Biological bulletin.

[B29] Gao Q, Kripke KE, Saldanha AJ, Yan W, Holmes S, Small PM (2005). Gene expression diversity among Mycobacterium tuberculosis clinical isolates. Microbiology (Reading, England).

[B30] Sassetti CM, Rubin EJ (2003). Genetic requirements for mycobacterial survival during infection. Proc Natl Acad Sci USA.

[B31] Tuberculist Web Server, Pasteur Institute. http://genolist.pasteur.fr/TubercuList/.

[B32] Price ND, Reed JL, Palsson BO (2004). Genome-scale models of microbial cells: evaluating the consequences of constraints. Nat Rev Microbiol.

[B33] Papin JA, Reed JL, Palsson BO (2004). Hierarchical thinking in network biology: the unbiased modularization of biochemical networks. Trends Biochem Sci.

[B34] Thiele I, Price ND, Vo TD, Palsson BO (2005). Candidate metabolic network states in human mitochondria. Impact of diabetes, ischemia, and diet. J Biol Chem.

[B35] Burgard AP, Nikolaev EV, Schilling CH, Maranas CD (2004). Flux coupling analysis of genome-scale metabolic network reconstructions. Genome Res.

[B36] Jamshidi N, Palsson BO (2006). Systems biology of SNPs. Molecular systems biology [electronic resource].

[B37] Pfeiffer T, Sanchez-Valdenebro I, Nuno JC, Montero F, Schuster S (1999). METATOOL: for studying metabolic networks. Bioinformatics.

[B38] Palsson BO (2006). Systems Biology: Determining the Capabilities of Reconstructed Networks.

[B39] Mdluli K, Spigelman M (2006). Novel targets for tuberculosis drug discovery. Current opinion in pharmacology.

[B40] Systems Biology Research Group, UCSD. http://systemsbiology.ucsd.edu/.

[B41] Raman K, Rajagopalan P, Chandra N (2005). Flux balance analysis of mycolic Acid pathway: targets for anti-tubercular drugs. PLoS computational biology.

[B42] Reed JL, Famili I, Thiele I, Palsson BO (2006). Towards multidimensional genome annotation. Nat Rev Genet.

[B43] Hjersted JL, Henson MA, Mahadevan R (2007). Genome-scale analysis of Saccharomyces cerevisiae metabolism and ethanol production in fed-batch culture. Biotechnol Bioeng.

[B44] Van Dien SJ, Lidstrom ME (2002). Stoichiometric model for evaluating the metabolic capabilities of the facultative methylotroph Methylobacterium extorquens AM1, with application to reconstruction of C(3) and C(4) metabolism. Biotechnol Bioeng.

[B45] The Institute for Genomic Research. http://www.tigr.org/.

[B46] Kyoto Encyclopedia of Genes and Genomes. http://www.genome.jp/kegg/.

[B47] The SEED. http://theseed.uchicago.edu/FIG/index.cgi.

[B48] van Hemert PA, Tiesjema RH (1977). Possible use of the oxygen uptake rate in the evaluation of BCG vaccines. Journal of biological standardization.

