# Supplementary material for: Investigating the metabolic capabilities of Mycobacterium tuberculosis H37Rv using the in silico strain iNJ661 and proposing alternative drug targets
Source: BMC Syst Biol. 2007 Jun 8;1:26. doi: 10.1186/1752-0509-1-26 (PMC1925256; doi:10.1186/1752-0509-1-26)

Robustness plots (calculating the objective function as a function of different flux uptake rates) were compared between *iNJ*661 and *iJR*904 (*in silico E. Coli*). The *in silico Mtb* strain was grown on Middlebrook 7H9 media conditions and the *in silico E. Coli* strain was grown on aerobic glucose conditions. The phosphate and sulfate uptake rates were varied and the resulting biomass production rates were observed. The slope of the robustness plots, provide an approximation to the sensitivity of the objective function on the respective flux. The slope for phosphate was approximately the same between *iNJ*661 and *iJR*904 (~ 1), however it was about four times greater for sulfate in *iNJ*661 (~ 16) than *iJR*904 (~ 4).

iNJ661 Robustness Plots

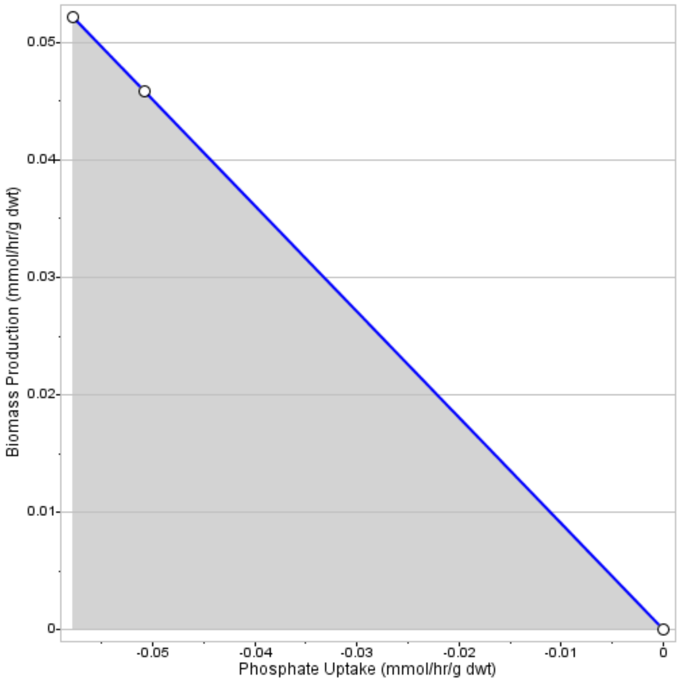

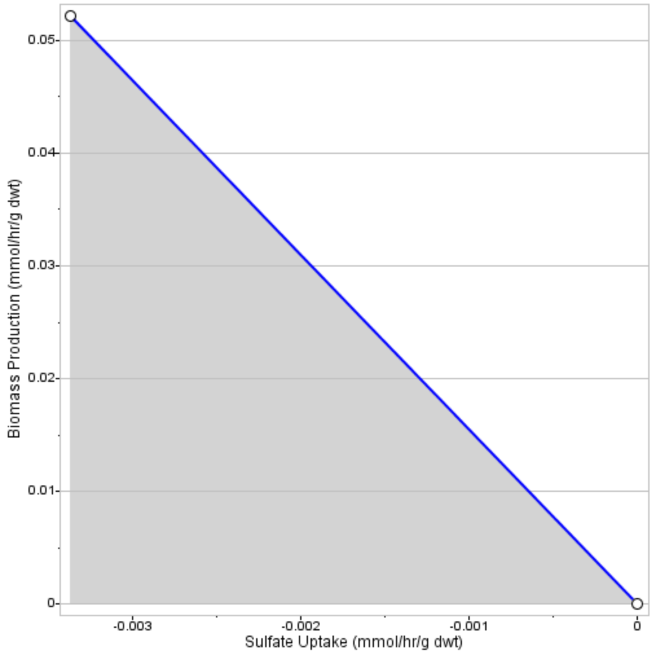

*iJR*904 Robustness Plots

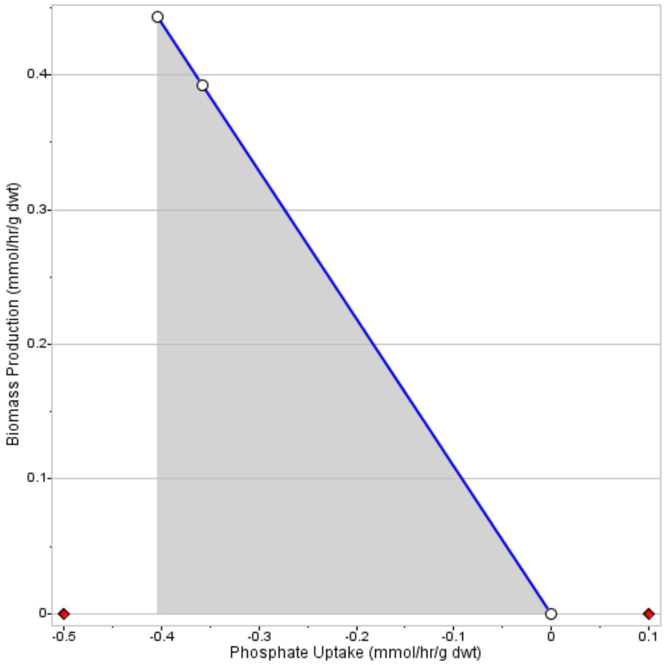

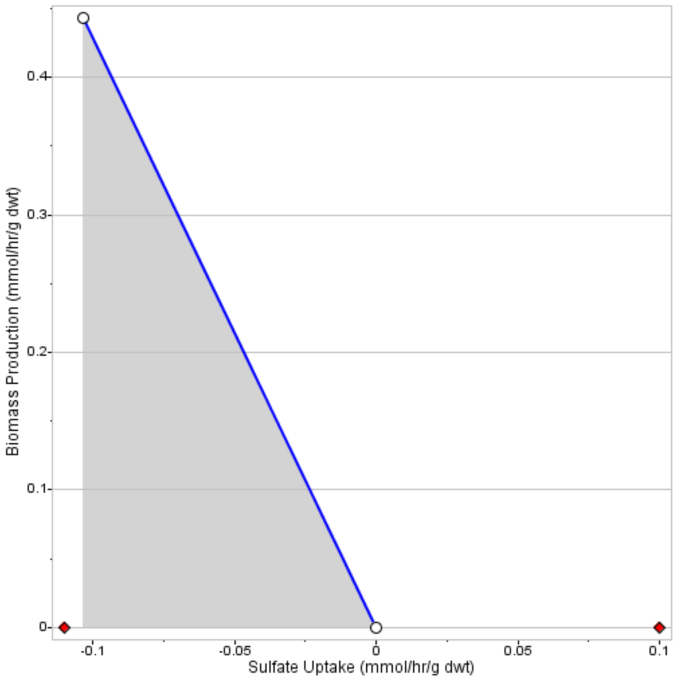

Supplement: Additional File 7 — Robustness plots for phosphate and sulfate for iNJ661 and iJR904. Plotting the biomass objective function versus phosphate or sulfate uptake rates for in silico strains of M. tb and E. coli. [file 1752-0509-1-26-S7.pdf]
