# Supplementary material for: Investigating the metabolic capabilities of Mycobacterium tuberculosis H37Rv using the in silico strain iNJ661 and proposing alternative drug targets
Source: BMC Syst Biol. 2007 Jun 8;1:26. doi: 10.1186/1752-0509-1-26 (PMC1925256; doi:10.1186/1752-0509-1-26)
Supplement: Additional File 6 — Explicit list of references used in the reconstruction process. The list of references used to define particular reactions and GPR relationships. [file 1752-0509-1-26-S6.pdf]

References used throughout the reconstruction (see notes field PMIDs)

Alderwick LJ, Seidel M, Sahm H, Besra GS, Eggeling L (2006) Identification of a novel arabinofuranosyltransferase (AftA) involved in cell wall arabinan biosynthesis in Mycobacterium tuberculosis. *J Biol Chem* **281:** 15653-15661.PMID: 16595677

Argyrou A, Blanchard JS (2004) Kinetic and chemical mechanism of Mycobacterium tuberculosis 1-deoxy-D-xylulose-5-phosphate isomeroreductase. *Biochemistry* **43:** 4375-4384.PMID: 15065882

Azad AK, Sirakova TD, Fernandes ND, Kolattukudy PE (1997) Gene knockout reveals a novel gene cluster for the synthesis of a class of cell wall lipids unique to pathogenic mycobacteria. *J Biol Chem* **272:** 16741-16745.PMID: 9201977

Bailey AM, Mahapatra S, Brennan PJ, Crick DC (2002) Identification, cloning, purification, and enzymatic characterization of Mycobacterium tuberculosis 1-deoxy-D-xylulose 5-phosphate synthase. *Glycobiology* **12:** 813-820.PMID: 12499403

Bertrand T, Eady NA, Jones JN, Jesmin, Nagy JM, Jamart-Gregoire B, Raven EL, Brown KA (2004) Crystal structure of Mycobacterium tuberculosis catalase-peroxidase. *J Biol Chem* **279:** 38991-38999.PMID: 15231843

Besra GS, Morehouse CB, Rittner CM, Waechter CJ, Brennan PJ (1997) Biosynthesis of mycobacterial lipoarabinomannan. *J Biol Chem* **272:** 18460-18466.PMID: 9218490

Bhatt AN, Shukla N, Aliverti A, Zanetti G, Bhakuni V (2005) Modulation of cooperativity in Mycobacterium tuberculosis NADPH-ferredoxin reductase: cation-and pH-induced alterations in native conformation and destabilization of the NADP+-binding domain. *Protein Sci* **14:** 980-992.PMID: 15741336

Boissier F, Bardou F, Guillet V, Uttenweiler-Joseph S, Daffe M, Quemard A, Mourey L (2006) Further insight into S-adenosylmethionine-dependent methyltransferases: structural characterization of Hma, an enzyme essential for the biosynthesis of oxygenated mycolic acids in Mycobacterium tuberculosis. *J Biol Chem* **281:** 4434-4445.PMID: 16356931

Braibant M, Gilot P, Content J (2000) The ATP binding cassette (ABC) transport systems of Mycobacterium tuberculosis. *FEMS Microbiol Rev* **24:** 449-467.PMID: 10978546

Brennan PJ, Nikaido H (1995) The envelope of mycobacteria. *Annu Rev Biochem* **64:** 29-63.PMID: 7574484

Briken V, Porcelli SA, Besra GS, Kremer L (2004) Mycobacterial lipoarabinomannan and related lipoglycans: from biogenesis to modulation of the immune response. *Mol Microbiol* **53:** 391-403.PMID: 15228522

Choi KH, Kremer L, Besra GS, Rock CO (2000) Identification and substrate specificity of beta - ketoacyl (acyl carrier protein) synthase III (mtFabH) from Mycobacterium tuberculosis. *J Biol Chem* **275:** 28201-28207.PMID: 10840036

Cole S (2005) *Tuberculosis and the tubercle bacillus*. ASM Press, Washington, DC.

Crick DC, Mahapatra S, Brennan PJ (2001) Biosynthesis of the arabinogalactan-peptidoglycan complex of Mycobacterium tuberculosis. *Glycobiology* **11:** 107R-118R.PMID: 11555614

Crick DC, Schulbach MC, Zink EE, Macchia M, Barontini S, Besra GS, Brennan PJ (2000) Polyprenyl phosphate biosynthesis in Mycobacterium tuberculosis and Mycobacterium smegmatis. *J Bacteriol* **182:** 5771-5778.PMID: 11004176

Dayaram YK, Talaue MT, Connell ND, Venketaraman V (2006) Characterization of a glutathione metabolic mutant of Mycobacterium tuberculosis and its resistance to glutathione and nitrosoglutathione. *J Bacteriol* **188:** 1364-1372.PMID: 16452418

De Smet KA, Weston A, Brown IN, Young DB, Robertson BD (2000) Three pathways for trehalose biosynthesis in mycobacteria. *Microbiology* **146 (Pt 1):** 199-208.PMID: 10658666

De Voss JJ, Rutter K, Schroeder BG, Barry CE, 3rd (1999) Iron acquisition and metabolism by mycobacteria. *J Bacteriol* **181:** 4443-4451.PMID: 10419938

Dhiman RK, Schaeffer ML, Bailey AM, Testa CA, Scherman H, Crick DC (2005) 1-Deoxy-D-xylulose 5-phosphate reductoisomerase (IspC) from Mycobacterium tuberculosis: towards understanding mycobacterial resistance to fosmidomycin. *J Bacteriol* **187:** 8395-8402.PMID: 16321944

Dubey VS, Sirakova TD, Cynamon MH, Kolattukudy PE (2003) Biochemical function of msl5 (pks8 plus pks17) in Mycobacterium tuberculosis H37Rv: biosynthesis of monomethyl branched unsaturated fatty acids. *J Bacteriol* **185:** 4620-4625.PMID: 12867474

Eady NA, Jesmin NA, Servos S, Cass AE, Nagy JM, Brown KA (2005) Probing the function of Mycobacterium tuberculosis catalase-peroxidase by site-directed mutagenesis. *Dalton Trans*: 3495-3500.PMID: 16315359

Faldt J, Dahlgren C, Karlsson A, Ahmed AM, Minnikin DE, Ridell M (1999) Activation of human neutrophils by mycobacterial phenolic glycolipids. *Clin Exp Immunol* **118:** 253-260.PMID: 10540187

Fischer F, Raimondi D, Aliverti A, Zanetti G (2002) Mycobacterium tuberculosis FprA, a novel bacterial NADPH-ferredoxin reductase. *Eur J Biochem* **269:** 3005-3013.PMID: 12071965

Gago G, Kurth D, Diacovich L, Tsai SC, Gramajo H (2006) Biochemical and structural characterization of an essential acyl coenzyme A carboxylase from Mycobacterium tuberculosis. *J Bacteriol* **188:** 477-486.PMID: 16385038

Gardner PR (1996) Superoxide production by the mycobacterial and pseudomonad quinoid pigments phthiocol and pyocyanine in human lung cells. *Arch Biochem Biophys* **333:** 267-274.PMID: 8806780

Ghiladi RA, Knudsen GM, Medzihradszky KF, Ortiz de Montellano PR (2005) The Met-Tyr-Trp cross-link in Mycobacterium tuberculosis catalase-peroxidase (KatG): autocatalytic formation and effect on enzyme catalysis and spectroscopic properties. *J Biol Chem* **280:** 22651-22663.PMID: 15840564

Gobin J, Moore CH, Reeve JR, Jr., Wong DK, Gibson BW, Horwitz MA (1995) Iron acquisition by Mycobacterium tuberculosis: isolation and characterization of a family of iron-binding exochelins. *Proc Natl Acad Sci U S A* **92:** 5189-5193.PMID: 7761471

Gurcha SS, Baulard AR, Kremer L, Locht C, Moody DB, Muhlecker W, Costello CE, Crick DC, Brennan PJ, Besra GS (2002) Ppm1, a novel polyprenol monophosphomannose synthase from Mycobacterium tuberculosis. *Biochem J* **365:** 441-450.PMID: 11931640

Huang H, Scherman MS, D'Haeze W, Vereecke D, Holsters M, Crick DC, McNeil MR (2005) Identification and active expression of the Mycobacterium tuberculosis gene encoding 5-phospho-{alpha}-d-ribose-1-diphosphate: decaprenyl-phosphate 5-phosphoribosyltransferase, the first enzyme committed to decaprenylphosphoryl-d-arabinose synthesis. *J Biol Chem* **280:** 24539-24543.PMID: 15878857

Jackson M, Crick DC, Brennan PJ (2000) Phosphatidylinositol is an essential phospholipid of mycobacteria. *J Biol Chem* **275:** 30092-30099.PMID: 10889206

James BW, Williams A, Marsh PD (2000) The physiology and pathogenicity of Mycobacterium tuberculosis grown under controlled conditions in a defined medium. *J Appl Microbiol* **88:** 669-677.PMID: 10792526

Johnston JM, Arcus VL, Baker EN (2005) Structure of naphthoate synthase (MenB) from Mycobacterium tuberculosis in both native and product-bound forms. *Acta Crystallogr D Biol Crystallogr* **61:** 1199-1206.PMID: 16131752

Johnston JM, Arcus VL, Morton CJ, Parker MW, Baker EN (2003) Crystal structure of a putative methyltransferase from Mycobacterium tuberculosis: misannotation of a genome clarified by protein structural analysis. *J Bacteriol* **185:** 4057-4065.PMID: 12837779

Kaur D, Brennan PJ, Crick DC (2004) Decaprenyl diphosphate synthesis in Mycobacterium tuberculosis. *J Bacteriol* **186:** 7564-7570.PMID: 15516568

Khuller GK, Taneja R, Kaur S, Verma JN (1982) Lipid composition and virulence of Mycobacterium tuberculosis H37Rv. *Aust J Exp Biol Med Sci* **60 (Pt 5):** 541-547.PMID: 6819845

King GM (2003) Uptake of carbon monoxide and hydrogen at environmentally relevant concentrations by mycobacteria. *Appl Environ Microbiol* **69:** 7266-7272.PMID: 14660375

Kordulakova J, Gilleron M, Puzo G, Brennan PJ, Gicquel B, Mikusova K, Jackson M (2003) Identification of the required acyltransferase step in the biosynthesis of the phosphatidylinositol mannosides of mycobacterium species. *J Biol Chem* **278:** 36285-36295.PMID: 12851411

Kremer L, Dover LG, Morehouse C, Hitchin P, Everett M, Morris HR, Dell A, Brennan PJ, McNeil MR, Flaherty C, Duncan K, Besra GS (2001) Galactan biosynthesis in Mycobacterium tuberculosis. Identification of a bifunctional UDP-galactofuranosyltransferase. *J Biol Chem* **276:** 26430-26440.PMID: 11304545

Lamichhane G, Zignol M, Blades NJ, Geiman DE, Dougherty A, Grosset J, Broman KW, Bishai WR (2003) A postgenomic method for predicting essential genes at subsaturation levels of mutagenesis: application to Mycobacterium tuberculosis. *Proc Natl Acad Sci U S A* **100:** 7213-7218.PMID: 12775759

LeMagueres P, Im H, Ebalunode J, Strych U, Benedik MJ, Briggs JM, Kohn H, Krause KL (2005) The 1.9 A crystal structure of alanine racemase from Mycobacterium tuberculosis contains a conserved entryway into the active site. *Biochemistry* **44:** 1471-1481.PMID: 15683232

Linder JU, Castro LI, Guo YL, Schultz JE (2004) Functional chimeras between the catalytic domains of the mycobacterial adenylyl cyclase Rv1625c and a Paramecium guanylyl cyclase. *FEBS Lett* **568:** 151-154.PMID: 15196937

Long MC, Escuyer V, Parker WB (2003) Identification and characterization of a unique adenosine kinase from Mycobacterium tuberculosis. *J Bacteriol* **185:** 6548-6555.PMID: 14594827

Mahapatra S, Yagi T, Belisle JT, Espinosa BJ, Hill PJ, McNeil MR, Brennan PJ, Crick DC (2005) Mycobacterial lipid II is composed of a complex mixture of modified muramyl and peptide moieties linked to decaprenyl phosphate. *J Bacteriol* **187:** 2747-2757.PMID: 15805521

Malathi VG, Ramakrishnan T (1966) Biosynthesis of nucleic acid purines in Mycobacterium tuberculosis H37Rv. *Biochem J* **98:** 594-597.PMID: 4957443

Mathur D, Ahsan Z, Tiwari M, Garg LC (2005) Biochemical characterization of recombinant phosphoglucose isomerase of Mycobacterium tuberculosis. *Biochem Biophys Res Commun* **337:** 626-632.PMID: 16212940

Mathur M, Kolattukudy PE (1992) Molecular cloning and sequencing of the gene for mycocerosic acid synthase, a novel fatty acid elongating multifunctional enzyme, from Mycobacterium tuberculosis var. bovis Bacillus Calmette-Guerin. *J Biol Chem* **267:** 19388-19395.PMID: 1527058

McCarthy AA, Peterson NA, Knijff R, Baker EN (2004) Crystal structure of MshB from Mycobacterium tuberculosis, a deacetylase involved in mycothiol biosynthesis. *J Mol Biol* **335:** 1131-1141.PMID: 14698305

McLean KJ, Clift D, Lewis DG, Sabri M, Balding PR, Sutcliffe MJ, Leys D, Munro AW (2006) The preponderance of P450s in the Mycobacterium tuberculosis genome. *Trends Microbiol* **14:** 220-228.PMID: 16581251

McLean KJ, Scrutton NS, Munro AW (2003) Kinetic, spectroscopic and thermodynamic characterization of the Mycobacterium tuberculosis adrenodoxin reductase homologue FprA. *Biochem J* **372:** 317-327.PMID: 12614197

Mikusova K, Huang H, Yagi T, Holsters M, Vereecke D, D'Haeze W, Scherman MS, Brennan PJ, McNeil MR, Crick DC (2005) Decaprenylphosphoryl arabinofuranose, the donor of the D-arabinofuranosyl residues of mycobacterial arabinan, is formed via a two-step epimerization of decaprenylphosphoryl ribose. *J Bacteriol* **187:** 8020-8025.PMID: 16291675

Mikusova K, Yagi T, Stern R, McNeil MR, Besra GS, Crick DC, Brennan PJ (2000) Biosynthesis of the galactan component of the mycobacterial cell wall. *J Biol Chem* **275:** 33890-33897.PMID: 10934214

Minnikin DE, Kremer L, Dover LG, Besra GS (2002) The methyl-branched fortifications of Mycobacterium tuberculosis. *Chem Biol* **9:** 545-553.PMID: 12031661

Morita YS, Patterson JH, Billman-Jacobe H, McConville MJ (2004) Biosynthesis of mycobacterial phosphatidylinositol mannosides. *Biochem J* **378:** 589-597.PMID: 14627436

Mougous JD, Petzold CJ, Senaratne RH, Lee DH, Akey DL, Lin FL, Munchel SE, Pratt MR, Riley LW, Leary JA, Berger JM, Bertozzi CR (2004) Identification, function and structure of the mycobacterial sulfotransferase that initiates sulfolipid-1 biosynthesis. *Nat Struct Mol Biol* **11:** 721-729.PMID: 15258569

Newton GL, Av-Gay Y, Fahey RC (2000a) A novel mycothiol-dependent detoxification pathway in mycobacteria involving mycothiol S-conjugate amidase. *Biochemistry* **39:** 10739-10746.PMID: 10978158

Newton GL, Av-Gay Y, Fahey RC (2000b) N-Acetyl-1-D-myo-inosityl-2-amino-2-deoxy-alpha-D-glucopyranoside deacetylase (MshB) is a key enzyme in mycothiol biosynthesis. *J Bacteriol* **182:** 6958-6963.PMID: 11092856

Newton GL, Fahey RC (2002) Mycothiol biochemistry. *Arch Microbiol* **178:** 388-394.PMID: 12420157

Newton GL, Koledin T, Gorovitz B, Rawat M, Fahey RC, Av-Gay Y (2003) The glycosyltransferase gene encoding the enzyme catalyzing the first step of mycothiol biosynthesis (mshA). *J Bacteriol* **185:** 3476-3479.PMID: 12754249

Nigou J, Dover LG, Besra GS (2002) Purification and biochemical characterization of Mycobacterium tuberculosis SuhB, an inositol monophosphatase involved in inositol biosynthesis. *Biochemistry* **41:** 4392-4398.PMID: 11914086

Nigou J, Gilleron M, Puzo G (2003) Lipoarabinomannans: from structure to biosynthesis. *Biochimie* **85:** 153-166.PMID: 12765785

Overbeek R, Begley T, Butler RM, Choudhuri JV, Chuang HY, Cohoon M, de Crecy-Lagard V, Diaz N, Disz T, Edwards R, Fonstein M, Frank ED, Gerdes S, Glass EM, Goesmann A, Hanson A, Iwata-Reuyl D, Jensen R, Jamshidi N, Krause L, et al. (2005) The subsystems approach to genome annotation and its use in the project to annotate 1000 genomes. *Nucleic Acids Res* **33:** 5691-5702.PMID: 16214803

Parish T, Schaeffer M, Roberts G, Duncan K (2005) HemZ is essential for heme biosynthesis in Mycobacterium tuberculosis. *Tuberculosis (Edinb)* **85:** 197-204.PMID: 15850757

Park SW, Hwang EH, Park H, Kim JA, Heo J, Lee KH, Song T, Kim E, Ro YT, Kim SW, Kim YM (2003) Growth of mycobacteria on carbon monoxide and methanol. *J Bacteriol* **185:** 142-147.PMID: 12486050

Patel MP, Blanchard JS (1999) Expression, purification, and characterization of Mycobacterium tuberculosis mycothione reductase. *Biochemistry* **38:** 11827-11833.PMID: 10512639

Penninckx MJ, Elskens MT (1993) Metabolism and functions of glutathione in micro-organisms. *Adv Microb Physiol* **34:** 239-301.PMID: 8095770

Perez E, Constant P, Laval F, Lemassu A, Laneelle MA, Daffe M, Guilhot C (2004a) Molecular dissection of the role of two methyltransferases in the biosynthesis of phenolglycolipids and phthiocerol dimycoserosate in the Mycobacterium tuberculosis complex. *J Biol Chem* **279:** 42584-42592.PMID: 15292265

Perez E, Constant P, Lemassu A, Laval F, Daffe M, Guilhot C (2004b) Characterization of three glycosyltransferases involved in the biosynthesis of the phenolic glycolipid antigens from the Mycobacterium tuberculosis complex. *J Biol Chem* **279:** 42574-42583.PMID: 15292272

Quadri LE, Sello J, Keating TA, Weinreb PH, Walsh CT (1998) Identification of a Mycobacterium tuberculosis gene cluster encoding the biosynthetic enzymes for assembly of the virulence-conferring siderophore mycobactin. *Chem Biol* **5:** 631-645.PMID: 9831524

Rainwater DL, Kolattukudy PE (1985) Fatty acid biosynthesis in Mycobacterium tuberculosis var. bovis Bacillus Calmette-Guerin. Purification and characterization of a novel fatty acid synthase,

mycocerosic acid synthase, which elongates n-fatty acyl-CoA with methylmalonyl-CoA. *J Biol Chem* **260:** 616-623.PMID: 3880746

Rao A, Ranganathan A (2004) Interaction studies on proteins encoded by the phthiocerol dimycocerosate locus of Mycobacterium tuberculosis. *Mol Genet Genomics* **272:** 571-579.PMID: 15668773

Ratledge C (2004) Iron, mycobacteria and tuberculosis. *Tuberculosis (Edinb)* **84:** 110-130.PMID: 14670352

Raymond JB, Mahapatra S, Crick DC, Pavelka MS, Jr. (2005) Identification of the namH gene, encoding the hydroxylase responsible for the N-glycolylation of the mycobacterial peptidoglycan. *J Biol Chem* **280:** 326-333.PMID: 15522883

Rhee KH, Lee KS, Priyadarshi A, Kim EE, Hwang KY (2005) Crystallization and preliminary X-ray crystallographic studies of fatty acid-CoA racemase from Mycobacterium tuberculosis H37Rv. *Acta Crystallograph Sect F Struct Biol Cryst Commun* **61:** 1017-1019.PMID: 16511223

Rivera-Marrero CA, Ritzenthaler JD, Newburn SA, Roman J, Cummings RD (2002) Molecular cloning and expression of a novel glycolipid sulfotransferase in Mycobacterium tuberculosis. *Microbiology* **148:** 783-792.PMID: 11882713

Rom W, Garay S (2004) *Tuberculosis*. Lippincott Williams & Wilkins, Philadelphia.

Rosenkrands I, King A, Weldingh K, Moniatte M, Moertz E, Andersen P (2000) Towards the proteome of Mycobacterium tuberculosis. *Electrophoresis* **21:** 3740-3756.PMID: 11271494

Sareen D, Steffek M, Newton GL, Fahey RC (2002) ATP-dependent L-cysteine:1D-myo-inosityl 2-amino-2-deoxy-alpha-D-glucopyranoside ligase, mycothiol biosynthesis enzyme MshC, is related to class I cysteinyl-tRNA synthetases. *Biochemistry* **41:** 6885-6890.PMID: 12033919

Sassetti CM, Boyd DH, Rubin EJ (2003) Genes required for mycobacterial growth defined by high density mutagenesis. *Mol Microbiol* **48:** 77-84.PMID: 12657046

Scherman MS, Kalbe-Bournonville L, Bush D, Xin Y, Deng L, McNeil M (1996) Polyprenylphosphate-pentoses in mycobacteria are synthesized from 5-phosphoribose pyrophosphate. *J Biol Chem* **271:** 29652-29658.PMID: 8939897

Schulbach MC, Brennan PJ, Crick DC (2000) Identification of a short (C15) chain Z-isoprenyl diphosphate synthase and a homologous long (C50) chain isoprenyl diphosphate synthase in Mycobacterium tuberculosis. *J Biol Chem* **275:** 22876-22881.PMID: 10816587

Sharma V, Sharma S, Hoener zu Bentrup K, McKinney JD, Russell DG, Jacobs WR, Jr., Sacchettini JC (2000) Structure of isocitrate lyase, a persistence factor of Mycobacterium tuberculosis. *Nat Struct Biol* **7:** 663-668.PMID: 10932251

Shenoy AR, Sreenath N, Podobnik M, Kovacevic M, Visweswariah SS (2005a) The Rv0805 gene from Mycobacterium tuberculosis encodes a 3',5'-cyclic nucleotide phosphodiesterase: biochemical and mutational analysis. *Biochemistry* **44:** 15695-15704.PMID: 16313172

Shenoy AR, Sreenath NP, Mahalingam M, Visweswariah SS (2005b) Characterization of phylogenetically distant members of the adenylate cyclase family from mycobacteria: Rv1647 from Mycobacterium tuberculosis and its orthologue ML1399 from M. leprae. *Biochem J* **387:** 541-551.PMID: 15500449

Sirakova TD, Dubey VS, Cynamon MH, Kolattukudy PE (2003) Attenuation of Mycobacterium tuberculosis by disruption of a mas-like gene or a chalcone synthase-like gene, which causes deficiency in dimycocerosyl phthiocerol synthesis. *J Bacteriol* **185:** 2999-3008.PMID: 12730158

Smith CV, Huang CC, Miczak A, Russell DG, Sacchettini JC, Honer zu Bentrup K (2003) Biochemical and structural studies of malate synthase from Mycobacterium tuberculosis. *J Biol Chem* **278:** 1735-1743.PMID: 12393860

Srinivasan V, Morowitz HJ (2006) Ancient genes in contemporary persistent microbial pathogens. *Biol Bull* **210:** 1-9.PMID: 16501059

Stadthagen G, Kordulakova J, Griffin R, Constant P, Bottova I, Barilone N, Gicquel B, Daffe M, Jackson M (2005) p-Hydroxybenzoic acid synthesis in Mycobacterium tuberculosis. *J Biol Chem* **280:** 40699-40706.PMID: 16210318

Steffek M, Newton GL, Av-Gay Y, Fahey RC (2003) Characterization of Mycobacterium tuberculosis mycothiol S-conjugate amidase. *Biochemistry* **42:** 12067-12076.PMID: 14556638

Stermann M, Bohrssen A, Diephaus C, Maass S, Bange FC (2003) Polymorphic nucleotide within the promoter of nitrate reductase (NarGHJI) is specific for Mycobacterium tuberculosis. *J Clin Microbiol* **41:** 3252-3259.PMID: 12843072

Sulzenbacher G, Canaan S, Bordat Y, Neyrolles O, Stadthagen G, Roig-Zamboni V, Rauzier J, Maurin D, Laval F, Daffe M, Cambillau C, Gicquel B, Bourne Y, Jackson M (2006) LppX is a lipoprotein required for the translocation of phthiocerol dimycocerosates to the surface of Mycobacterium tuberculosis. *Embo J* **25:** 1436-1444.PMID: 16541102

Takahashi S, Kuzuyama T, Watanabe H, Seto H (1998) A 1-deoxy-D-xylulose 5-phosphate reductoisomerase catalyzing the formation of 2-C-methyl-D-erythritol 4-phosphate in an alternative nonmevalonate pathway for terpenoid biosynthesis. *Proc Natl Acad Sci U S A* **95:** 9879-9884.PMID: 9707569

Telenti A, Philipp WJ, Sreevatsan S, Bernasconi C, Stockbauer KE, Wieles B, Musser JM, Jacobs WR, Jr. (1997) The emb operon, a gene cluster of Mycobacterium tuberculosis involved in resistance to ethambutol. *Nat Med* **3:** 567-570.PMID: 9142129

Tian J, Bryk R, Itoh M, Suematsu M, Nathan C (2005a) Variant tricarboxylic acid cycle in Mycobacterium tuberculosis: identification of alpha-ketoglutarate decarboxylase. *Proc Natl Acad Sci U S A* **102:** 10670-10675.PMID: 16027371

Tian J, Bryk R, Shi S, Erdjument-Bromage H, Tempst P, Nathan C (2005b) Mycobacterium tuberculosis appears to lack alpha-ketoglutarate dehydrogenase and encodes pyruvate dehydrogenase in widely separated genes. *Mol Microbiol* **57:** 859-868.PMID: 16045627

Truglio JJ, Theis K, Feng Y, Gajda R, Machutta C, Tonge PJ, Kisker C (2003) Crystal structure of Mycobacterium tuberculosis MenB, a key enzyme in vitamin K2 biosynthesis. *J Biol Chem* **278:** 42352-42360.PMID: 12909628

Vetting MW, Roderick SL, Yu M, Blanchard JS (2003) Crystal structure of mycothiol synthase (Rv0819) from Mycobacterium tuberculosis shows structural homology to the GNAT family of N-acetyltransferases. *Protein Sci* **12:** 1954-1959.PMID: 12930994

Wheeler PR, Coldham NG, Keating L, Gordon SV, Wooff EE, Parish T, Hewinson RG (2005) Functional demonstration of reverse transsulfuration in the Mycobacterium tuberculosis complex reveals that methionine is the preferred sulfur source for pathogenic Mycobacteria. *J Biol Chem* **280:** 8069-8078.PMID: 15576367

Wolucka BA, McNeil MR, de Hoffmann E, Chojnacki T, Brennan PJ (1994) Recognition of the lipid intermediate for arabinogalactan/arabinomannan biosynthesis and its relation to the mode of action of ethambutol on mycobacteria. *J Biol Chem* **269:** 23328-23335.PMID: 8083238

Wooff E, Michell SL, Gordon SV, Chambers MA, Bardarov S, Jacobs WR, Jr., Hewinson RG, Wheeler PR (2002) Functional genomics reveals the sole sulphate transporter of the Mycobacterium tuberculosis complex and its relevance to the acquisition of sulphur in vivo. *Mol Microbiol* **43:** 653-663.PMID: 11929522
